# A pH and Redox Dual Responsive 4-Arm Poly(ethylene glycol)-block-poly(disulfide histamine) Copolymer for Non-Viral Gene Transfection *in Vitro* and *in Vivo*

**DOI:** 10.3390/ijms15059067

**Published:** 2014-05-21

**Authors:** Kangkang An, Peng Zhao, Chao Lin, Hongwei Liu

**Affiliations:** 1 Department of Periodontoloy, Laboratory of Oral Biomedical Science and Translational Medicine, School of Stomatology, Tongji University, Shanghai 200072, China; E-Mail: xxstiger@tongji.edu.cn; 2 Institute of Biomedical Engineering and Nanoscience, Tongji University School of Medicine, Tongji University, Shanghai 200092, China; E-Mail: tjzhaopeng@gmail.com

**Keywords:** poly(disulfide histamine), pH targeting, gene delivery, pH-responsive, cancer cells

## Abstract

A novel 4-arm poly(ethylene glycol)-b-poly(disulfide histamine) copolymer was synthesized by Michael addition reaction of poly(ethylene glycol) (PEG) vinyl sulfone and amine-capped poly(disulfide histamine) oligomer, being denoted as 4-arm PEG-SSPHIS. This copolymer was able to condense DNA into nanoscale polyplexes (<200 nm in average diameter) with almost neutral surface charge (+(5–10) mV). Besides, these polyplexes were colloidal stable within 4 h in HEPES buffer saline at pH 7.4 (physiological environment), but rapidly dissociated to liberate DNA in the presence of 10 mM glutathione (intracellular reducing environment). The polyplexes also revealed pH-responsive surface charges which markedly increased with reducing pH values from 7.4–6.3 (tumor microenvironment). *In vitro* transfection experiments showed that polyplexes of 4-arm PEG-SSPHIS were capable of exerting enhanced transfection efficacy in MCF-7 and HepG2 cancer cells under acidic conditions (pH 6.3–7.0). Moreover, intravenous administration of the polyplexes to nude mice bearing HepG2-tumor yielded high transgene expression largely in tumor rather other normal organs. Importantly, this copolymer and its polyplexes had low cytotoxicity against the cells *in vitro* and caused no death of the mice. The results of this study indicate that 4-arm PEG-SSPHIS has high potential as a dual responsive gene delivery vector for cancer gene therapy.

## 1. Introduction

Gene therapy presents a novel strategy for the treatment of various significant human diseases with gene defects such as cancer [1]. A key challenge for successful human gene therapy is the availability of safe and efficient gene delivery vectors. Although recombinant viral vectors have been employed widely in clinical gene therapy trials, their uncertain bio-safety issues such as oncogenicity, immunogenicity and cytotoxicity are dangerous hurdles to clinical application [2,3]. Alternatively, non-viral gene delivery vectors, for example, cationic polymers do not have these problems and take additional advantages of versatile molecular structure and handy modifications [4–6]. A lot of commercial cationic polymers such as polylysine and polyethylenimine have been found which can bind DNA to form polymer/DNA complexes (polyplexes) with nanoscale size and positively-charged surface charge, yielding detectable transfection activity *in vitro* [7,8]. However, their further clinical translation is hampered seriously by either low transfection efficacy or high cytotoxicity after repeated administration [5,6].

For high transfection efficacy with low cytotoxicity, in the past two decades, much effort has been made in the design of biodegradable cationic polymers for controlled gene delivery [9]. Particularly, disulfide-containing (bioreducible) cationic polymers have received much attention in recent years due to redox-responsive attribute of disulfide bond, that is, relatively chemically stable in an extracellular environment but degradable by glutathione (5–10 mM) in an intracellular reducing environment [10]. Thus, polyplexes of bioreducible cationic polymers are capable of redox-responsive unpacking by the disulfide cleavage and then efficient gene unloading inside the cells, thereby promoting transfection efficacy. Furthermore, this intracellular degradation process causes lower cytotoxicity for bioreducible cationic polymers compared to their non-degradable counterparts [11]. For these reasons, bioreducible cationic polymers have great potential as new-generation non-viral gene delivery vectors [12].

We and Engbersen have developed bioreducible poly(amido amine)s (SSPAAs) for efficient gene delivery against COS-7 cells *in vitro* [13,14]. Kim *et al*. reported on poly(disulfide amine)s for highly efficient gene transfection *in vitro* towards different cancer cells [12,15]. However, further utility of these bioreducible polymers for gene delivery *in vivo* is seriously limited because cationic polyplexes normally have poor colloidal stability and strong interactions with blood components, thus causing the formation of aggregates under physiological conditions [16,17]. Moreover, after intravenous injection, cationic polyplexes are rapidly eliminated by the reticuloendothelial system [18]. To overcome these problems, we modified SSPAAs with poly(ethylene glycol) (PEG) and found that PEGylated SSPAA-based polyplexes had enhanced colloidal stability and neutral surface charge, thus being suited for *in vivo* gene delivery [19]. However, these PEGylated polyplexes reveal inferior transfection ability to their unPEGylated polyplexes in MCF-7 cancer cells because neutral surface of PEGylated polyplexes impaired their cellular uptake [20]. Further studies are thus needed to address this “PEG dilemma”.

**Scheme 1.**
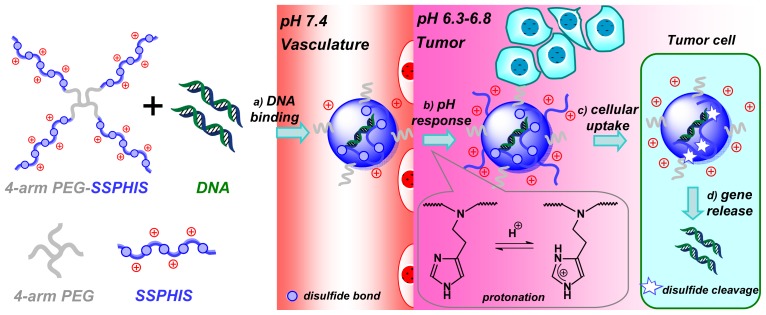
Schematic illustration of pH-targeting gene delivery towards tumor cells with 4-arm PEG-SSPHIS copolymer as a pH and redox dual responsive gene delivery vector: (**a**) gene binding of 4-arm PEG-SSPHIS copolymer to form the polyplexes with almost neutral surface; (**b**) increased surface charge upon the polyplexes in acidic tumor microenvironment via protonation of imidazole groups; (**c**) enhanced cellular uptake of the polyplexes to tumor cells; (**d**) intracellular gene release after disulfide cleavage.

For efficient *in vivo* gene delivery to tumor, PEG-ligand conjugation and PEG-deshielding are two major methods for overcoming “PEG dilemma” [21]. Besides, pH-targeting to acidic tumor is another practical method for tumor-targeted delivery. As such, a lot of pH-responsive polymeric micelles are developed for pH-targeting delivery of anti-cancer drugs towards tumor [22]. However, to the best of our knowledge, no report has yet appeared on cationic polymers for pH-targeting gene delivery. In this study, we designed and prepared 4-arm PEG-block-bioreducible poly(disulfide histamine) (denoted as 4-arm PEG-SSPHIS) copolymer for pH-targeting gene delivery (Scheme 1). It is hypothesized that the polyplexes of the copolymer have a neutral surface under physiological conditions, but positive surface in an acidic tumor microenvironment by the protonation of imidazole groups in histamine residues, thus inducing enhanced cellular uptake of the polyplexes in tumor cells. Further, intracellular cleavage of disulfide bond causes efficient gene unpacking of the polyplexes, thereby affording high transfection efficacy. Biophysical properties of 4-arm PEG-SSPHIS were characterized in terms of particle size, surface charge, gene binding and release as well as colloidal stability of polyplex. *In vitro* transfection activity and cytotoxicity of 4-arm PEG-SSPHIS were evaluated against different cancer cells at pH 7.4 and acidic pH values. Also, *in vivo* transfection efficiency induced by the polyplexes was evaluated by intravenous administration in HepG2-bearing nude mice.

## 2. Results and Discussion

### 2.1. Synthesis and Characterization of 4-Arm PEG-Conjugated Bioreducible Poly(disulfide histamine)

**Figure 1.**
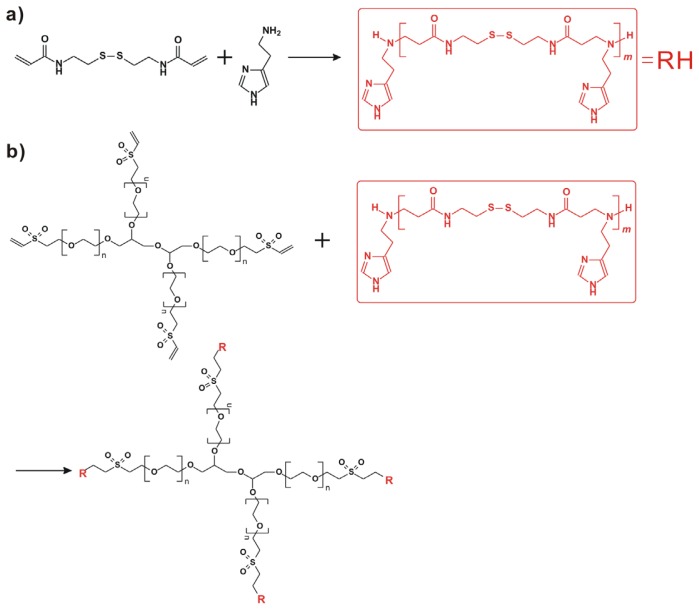
(**a**) Synthesis of poly(disulfide histamine) oligomer via Michael addition reaction of cystamine bisacrylamide and an excess amount of histamine; (**b**) Preparation of 4-arm PEG-b-poly(disulfide histamine) copolymer (denoted as 4-arm PEG-SSPHIS).

**Figure 2.**
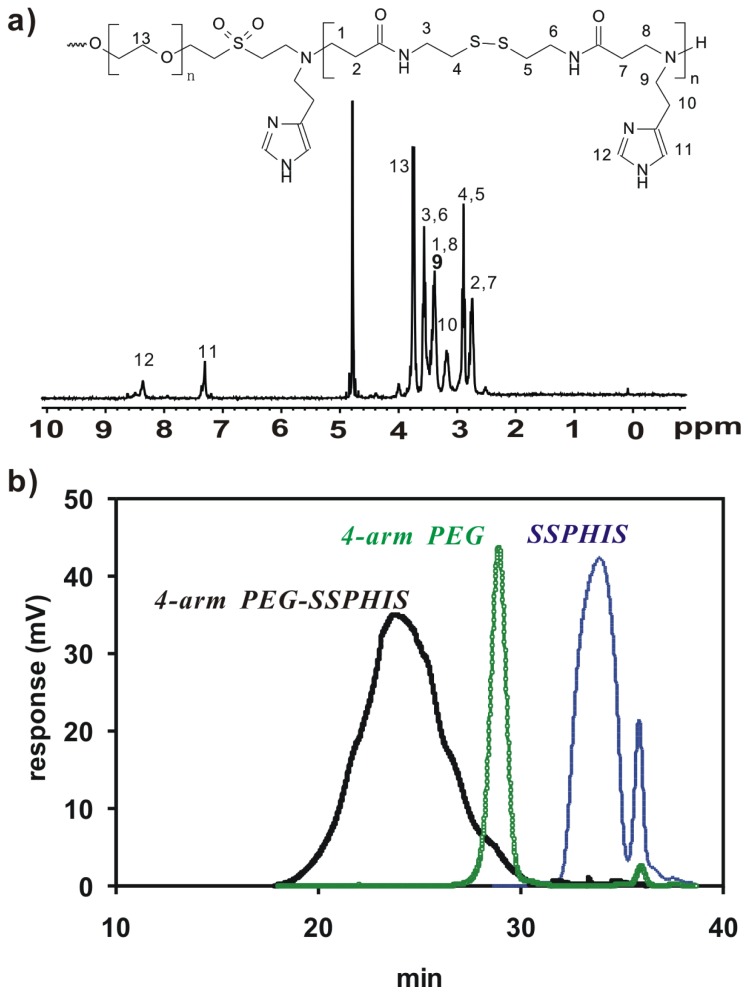
(**a**) ^1^H NMR spectra (D_2_O, 300 MHz) of 4-arm PEG-SSPHIS; (**b**) GPC chromatogram of 4-arm PEG-SSPHIS, 4-arm PEG and SSPHIS oligomer.

Herein, 4-arm PEG-b-poly(disulfide histamine) (denoted as 4-arm PEG-SSPHIS) copolymer was prepared via a two-step procedure (Figure 1). First, amino-terminated poly(disulfide histamine) (SSPHIS) oligomer was prepared by Michael addition reaction of cystamine bisacrylamide (CBA) and an excess amount of histamine (HIS). The number-average polymerization degree (*X_n_*) of the SSPHIS oligomer was modulated with a stoichiometric ratio (*r*) of the monomers, *i.e*., *r*_CBA/HIS_ = 0.8, which means that the *X_n_* of SSPHIS oligomer is 9 as calculated with the equation: *X_n_* = (1 + *r*)/(1 − *r*). Next, a 10-fold excess of SSPHIS oligomer was reacted with 4-arm PEG vinylsulfone by Michael addition reaction to ensure the formation of 4-arm PEG-SSPHIS copolymer and to minimize uncontrolled multiblock polymerisation reaction. Finally, 4-arm PEG-SSPHIS was purified by exhaustive hydrolysis (15,000 cut-off) with pH ~5 deionized water and finally obtained as its HCl-salt after freeze-drying. This copolymer had good solubility in 20 mM PBS and HEPES buffer at pH 7.4 at the concentration of 5 mg/mL. ^1^H NMR revealed that the composition of 4-arm PEG-SSPHIS was in accordance with its expected chemical structures (Figure 2a). Besides, no signals between 6 and 7 ppm was detected, suggesting that vinylsulfone group was consumed completely and 4-arm PEG was end-capped by SSPHIS residue. Furthermore, *ca*. ~40 HIS residues per 4-arm PEG-SSPHIS copolymer was determined by comparing the integrals of the peaks at δ 7.35 and 3.75, attributed to the proton (N=CH) in HIS residue and methylene protons (CH_2_CH_2_O) in PEG residue, respectively. This result means about 10 HIS-residues per SSPHIS chain, that is close to *X_n_* = 9 of SSPHIS oligomer, suggesting the formation of 4-arm PEG-SSPHIS copolymer. The weight-average molecular weight (*M_W_*) of the copolymer was measured using gel permeation chromatography (GPC). Figure 2b exhibits the typical GPC traces of the copolymer and starting reactants, 4-arm PEG and SSPHIS oligomer. The peak of this copolymer showed a unimodal distribution in its GPC trace with an earlier elution time (*M_W_* = 26.9 kDa) as compared to that of 4-arm PEG (*M_W_* = 10 kDa) and SSPHIS (*M_W_* = 5.2 kDa). Overall, GPC and NMR results indicate successful availability of 4-arm PEG-SSPHIS copolymer as well as the absence of SSPHIS oligomer and 4-arm PEG in this product.

### 2.2. Biophysical Properties of the Polyplexes of 4-Arm PEG-SSPHIS

**Figure 3.**
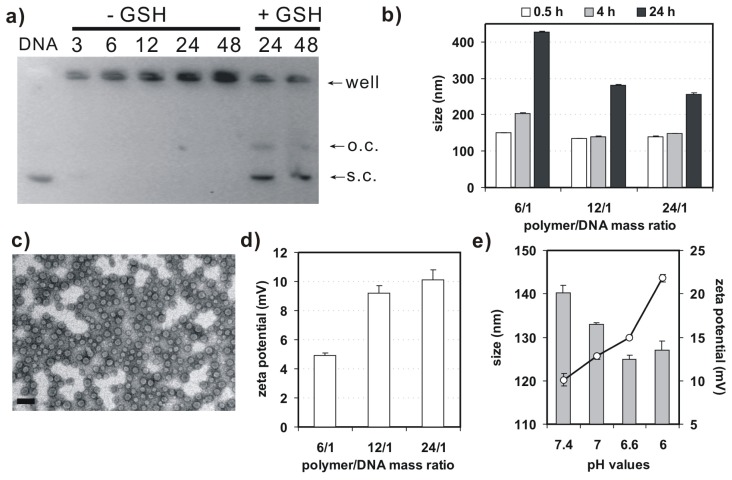
(**a**) Argarose gel retardation assay on binding behavior of 4-arm PEG-SSPHIS and plasmid DNA with (−GSH) or without (+GSH) of glutathione (GSH); (**b**) Particle size of the polyplexes of 4-arm PEG-SSPHIS at different polymer/DNA ratios as a function of incubation time in the presence of 130 mM NaCl; (**c**) Typical TEM image of polyplexes of 4-arm PEG-SSPHIS at the mass ratio of 12/1 (scale bar: 100 nm); (**d**) Zeta potential of the polyplexes of 4-arm PEG-SSPHIS at different polymer/DNA ratios; (**e**) Effect of pH values on size and zeta potential of the polyplexes of 4-arm PEG-SSPHIS at the mass ratio of 24/1.

Agarose gel retardation assay was performed to evaluate binding behavior of 4-arm PEG-SSPHIS copolymer and plasmid DNA (Figure 3a). This copolymer could efficiently retard the mobility of DNA at and above polymer/DNA mass ratios of 3/1, implying complexation between the copolymer and DNA. In previous studies, we found that disulfide-based poly(amido amine)s caused higher gene transfection efficacy as compared to their counterparts without the disulfide due to a facilitated DNA release from disulfide-based polyplexes by the disulfide cleavage in an intracellular reducing environment [13]. For this reason, DNA release from the polyplexes of 4-arm PEG-SSPHIS copolymer was investigated by the gel retardation assay in the presence of 10 mM glutathione, a thiol-containing peptide present in the cytoplasm. As expected, adequate DNA release from the polyplexes was clearly detected at the mass ratios of 24/1 and 48/1 (Figure 3a), indicating that the polyplexes may mediate a facilitated intracellular DNA release in the cellular interior.

Next, dynamic light scattering (DLS) analysis was applied to measure size and surface charge of 4-arm PEG-SSPHIS-based polyplexes. At the mass ratios in the range from 6/1–24/1, this copolymer could strongly bind DNA to form nanoscale polyplexes with average hydrodynamic sizes of ~135–150 nm (Figure 3b). In order to learn colloidal stability of the polyplexes, hydrodynamic size of 4-arm PEG-SSPHIS-based polyplexes was followed as a function of incubation time in pH 7.4 HEPES buffer (20 mM) with 130 mM NaCl, mimicking a physiological condition. It was found that the sizes of the polyplexes at the mass ratios maintained stable (<200 nm) at 4 h and gradually augmented to ~250–400 nm at 24 h, indicating that the polyplexes possess an enhanced colloidal stability (Figure 3b). Typical image of the polyplexes, observed under TEM, was exhibited in Figure 3c. These polyplexes had spherical shape with average particle sizes less than 100 nm. The sizes of the polyplexes determined by DLS analysis were bigger than those obtained by TEM, probably due to different state of the polyplexes. DLS analysis showed the size of polyplexes in a hydrated state, whereas TEM images exhibited the size at a dried state. As a result, the polyplexes had a larger hydrodynamic volume in the hydrated state due to the hydrophilic polymer coatings of the polyplexes and solvent effects. Furthermore, DLS analysis indicated that the polyplexes of 4-arm PEG-SSPHIS had the zeta-potentials in the range of +(5–10) mV (Figure 3d). This almost neutral surface is probably due to surface shielding of inner, positively-charged SSPHIS/DNA complexes by neutral 4-arm PEG chain.

Figure 3e presents particle size and surface charge of the polyplexes of 4-arm PEG-SSPHIS under an acidic condition, mimicking the microenvironment of solid tumor. It was shown that, with reducing pH values from 7.4–6.0, the sizes of the polyplexes slightly decreased from about 140–128 nm, but their surface charges markedly augmented from (+10)–(+22) mV. It was thought that, with reducing pH values, more and more imidazole groups (pK_a_~6.5) in the copolymer were protonated. As a result, the increased charge density of the copolymer caused reduced size and increased surface charge of the polyplexes. The results manifest that 4-arm PEG-SSPHIS may response to an acidic microenvironment of solid tumor. Overall, these gene delivery properties such as nanosized particle size, enhanced colloidal stability, and pH-responsive surface charge imply that 4-arm PEG-SSPHIS copolymer is favorable as a candidate for gene delivery towards cancer cells (*vide infra*).

### 2.3. 4-Arm PEG-SSPHIS Induces Efficient Gene Transfection in HepG2 Cancer Cells in Vitro

**Figure 4.**
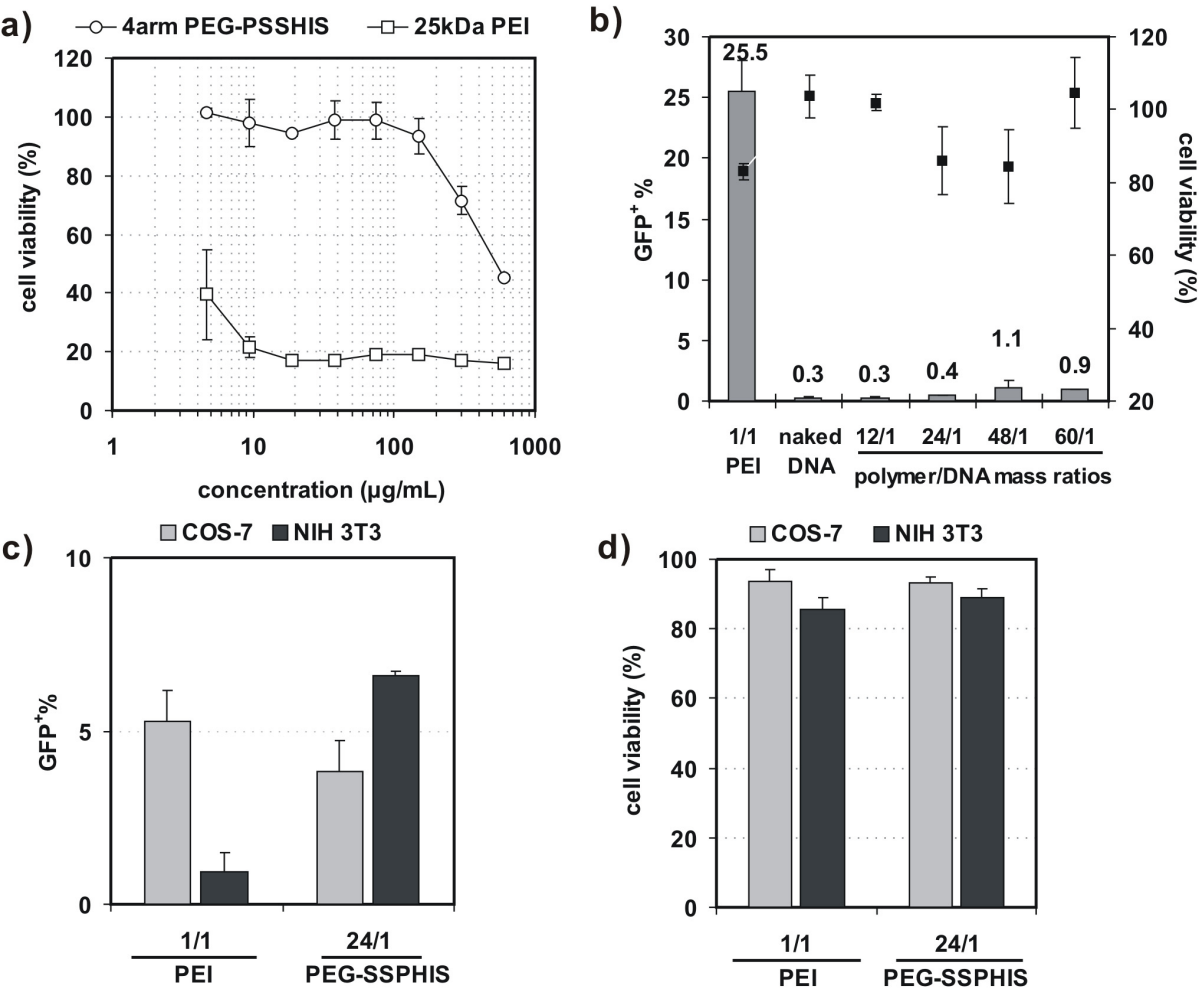
(**a**) Cytotoxicity of 4-arm PEG-SSPHIS copolymer (and 25 kDa PEI as a control) against COS-7 cells at different polymer concentrations; (**b**) Transfection efficiency and cytotoxicity of 4-arm PEG-SSPHIS in COS-7 cells at 48 h after 4 h co-incubation with the polyplexes of 4-arm PEG-SSPHIS at the mass ratio of 24/1 (2 μg DNA) in the absence of serum and then 44 h-post transfection. Polyplexes of 25 kDa PEI at an optimal ratio of 1/1 was used as a control; (**c**,**d**) Transfection efficiency (**c**) and cytotoxicity (**d**) of 4-arm PEG-SSPHIS in COS-7 and NIH 3T3 cells at 48 h after 4 h co-incubation with the polyplexes of 4-arm PEG-SSPHIS at the mass ratio of 24/1 (8 μg DNA) in the presence of serum and then 44 h-post transfection. Polyplexes of 25 kDa PEI at an optimal ratio of 1/1 was used as a control.

Before 4-arm PEG-SSPHIS copolymer is applied for gene transfection, it is essential to test whether the copolymer has an effect on cell viability. As such, the cytotoxicity of COS-7 cells was evaluated by AlamarBlue assay after the cells were co-incubated with the copolymer at varying concentrations from 5–600 μg/mL. This copolymer revealed low cytotoxicity with ~100% of cell viability at the polymer concentrations less than 150 μg/mL (Figure 4a). By contrast, 25 kDa PEI had extremely high cytotoxicity with only ~20%–40% of viable cells at the concentrations from 5–600 μg/mL. These results show that 4-arm PEG-SSPHIS copolymer has good cyto-biocompatibility.

*In vitro* transfection activity of 4-arm PEG-SSPHIS was evaluated against four cell lines (*i.e.*, COS-7, NIH 3T3, MCF-7 and HepG2) with plasmid DNA encoding green fluorescent protein (GFP) gene. Herein, COS-7 and NIH 3T3 cells were chosen as the models of normal tissue cells whereas MCF-7 and HepG2 cells as solid tumor cells. First, transfection efficiency of 4-arm PEG-SSPHIS was investigated as a function of polymer/DNA mass ratios in the absence of serum and presented as the percentage of GFP-expressing (GFP^+^) cells. As shown in Figure 4b, the polyplexes of 4-arm PEG-SSPHIS at different mass ratios from 12/1–60/1 failed to potently transfect COS-7 cells with low efficiencies (0.3%–1% GFP^+^). As a positive control, the polyplexes of 25 kDa-PEI afforded higher transfection efficiency (25.5% GFP^+^) at an optimal mass ratio of 1/1 [13,14]. Next, transfection activity of 4-arm PEG-SSPHIS was examined in the presence of 10% serum using a high DNA dose of 8 μg (Figure 4c). In this case, the polyplexes of 4-arm PEG-SSPHIS at the mass ratio of 24/1 again yielded low transfection efficiency against COS-7 and NIH 3T3 cells (3.8% and 6.6% GFP^+^ cells, respectively). A value of 25 kDa-PEI also induced low transfection efficacy (5.1% and 0.9% GFP^+^ cells, respectively). The inactive transfection is most likely attributed to the fact that negative serum adversely harms efficient cellular uptake of cationic polyplexes of the PEI. AlamarBlue assay showed that the polyplexes of 4-arm PEG-SSPHIS had low cytotoxicity with >80% cell viability (Figure 4b,d), revealing that 4-arm PEG-SSPHIS is harmless to the cells.

**Figure 5.**
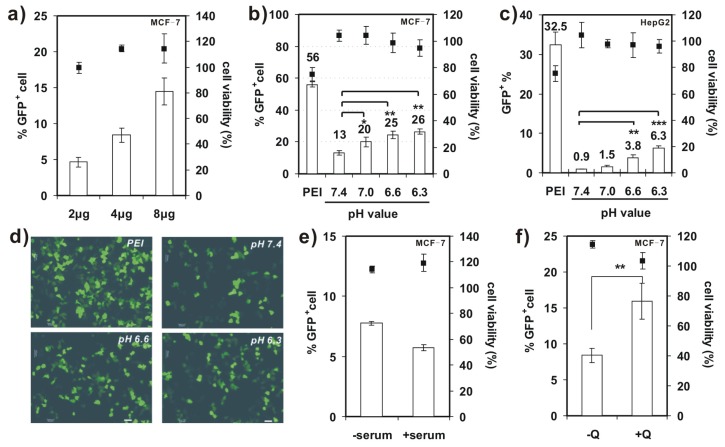
(**a**) Transfection efficiency and cytotoxicity of 4-arm PEG-SSPHIS in MCF-7 cells as a function of DNA dose (2–8 μg); (**b**,**c**) Transfection efficiency and cytotoxicity of 4-arm PEG-SSPHIS using 8 μg DNA against MCF-7 (**b**) and HepG2 (**c**) cells as a function of pH values. The cells were detected at 48 h after 4 h transfection with the polyplexes of 4-arm PEG-SSPHIS in the absence of serum and 44 h-post transfection. Polyplexes of 25 kDa PEI at the mass ratio of 1/1 was used as a control. * *p* < 0.05, ** *p* < 0.01, *** *p* < 0.001; (**d**) Typical images of GFP^+^ MCF-7 cells after transfection at varying pH values (scale bar: 50 nm); (**e**) Effect of FBS on the transfection efficiency of 4-arm PEG-SSPHIS with 4 μg of DNA; (**f**) Effect of 100 μM chloroquine (CQ) on the transfection efficiency of 4-arm PEG-SSPHIS with 4 μg of DNA. ** *p* < 0.01. All the polyplexes were prepared at the mass ratio of 12/1.

Further studies on transfection activity of 4-arm PEG-SSPHIS were conducted against MCF-7 and HepG2 cells at the mass ratio of 12/1 in the absence of serum. It was found that transfection efficiency of 4-arm PEG-SSPHIS in MCF-7 cells markedly increased from 4.6%–14.4% GFP^+^ when DNA dose was increased from 2–8 μg (Figure 5a). Moreover, with the 8 μg of DNA dose, the transfection efficiency of 4-arm PEG-SSPHIS increased to 26.5% GFP^+^ after lowering pH values from 7.4 (physiological pH) to 6.3 (tumor microenvironment pH) (Figure 5b). Similar phenomenon was also observed against HepG2 cells, where the efficiency of 4-arm PEG-SSPHIS markedly augmented by *ca*. 7.0 times (Figure 5c). Figure 5d exhibits typical images of GFP-expressing MCF-7 cells, observed under fluorescent microscopy, after the cells are transfected at different pH values. It can be seen clearly that larger amounts of GFP^+^ cells can be found at acidic pH values compared to the case at pH 7.4. Importantly, the active transfection activity was followed with ~100% cell viability (Figure 5a–c). However, a mild cytotoxicity (~75% cell viability) was observed for the polyplexes of 25 kDa PEI, although they yielded higher transfection efficiency, *i.e*., 56% and 32.5% GFP^+^ for MCF-7 and HepG2 cells, respectively. This high cytotoxicity is most likely because, with 8 μg of DNA, the PEI was applied at a high concentration (*ca*. 8 μg/mL) in transfection, thus causing cytotoxicity (Figure 4a).

We also evaluated the effect of serum on transfection activity of 4-arm PEG-SSPHIS copolymer in MCF-7 cells (Figure 5e). In the presence of 10% FBS, transfection efficiency of the copolymer reduced slightly from 7.8%–5.7% GFP^+^ cells when 4 μg of DNA was applied, suggesting that the FBS has a little effect on the transfection activity of 4-arm PEG-SSPHIS. Again, the polyplexes of 4-arm PEG-SSPHIS in the serum displayed low cytotoxicity with ~100% of viable cells.

A few previous studies showed that chloroquine (CQ), an endosomolytic agent, could enhance gene transfection activity by promoting endosomal disruption [23]. As indicated in Figure 5f, the addition of 100 μM CQ resulted in enhanced transfection activity of 4-arm PEG-SSPHIS-based polyplexes against MCF-7 cells (15.9% ± 2.5% with CQ *vs*. 8.4% ± 1% without CQ), meanwhile maintaining low cytotoxicity (~100% cell viability). This enhanced transfection activity may serve as a hint that the PEG shielding of the polyplexes hampers active interactions between cationic SSHIS residue with the endosomal membrane, and such interactions are critical for efficient endosomal escape of the polyplexes. This point was also supported by the findings from Wagner *et al*., who showed that the polyplexes of PEGylated PEIs yielded poor transfection efficiency as compared to those of unPEGylated PEIs, due to poor endosomal escape [24]. However, an incorporation of a pH-sensitive linker which can liberate PEG from PEI inside the endosomes could favorably enhance transfection efficiency [25]. Thus, further work may focus on the design of 4-arm PEG-linked SSPHIS that has a pH-liable linker between PEG and SSPHIS.

### 2.4. Transgene Expression Induced by Polyplexes of 4-Arm PEG-SSPHIS in HepG2 Tumor

**Figure 6.**
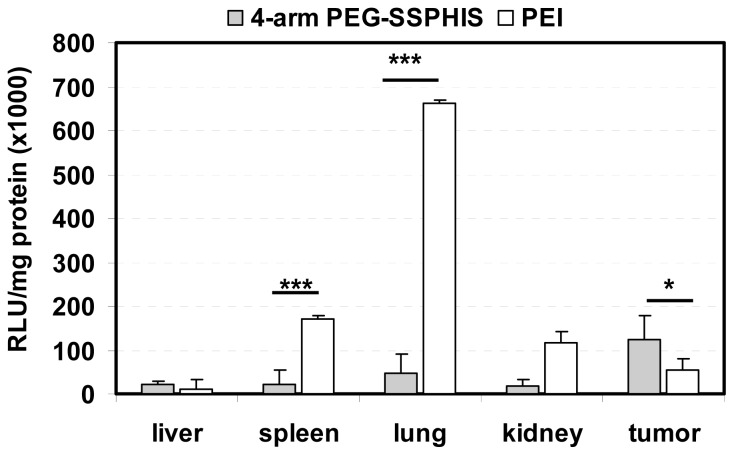
Luciferase expression in the tumor and organs of nude mice after intravenous injection of 4-arm PEG-SSPHIS-based polyplexes (30 μg DNA, at mass ratio of 12/1). The expression was presented as RLU/mg protein (*n* = 4). A formulation of PEI at an optimal mass ratio of 1/1 was used as a control. * *p* < 0.05, *** *p* < 0.001.

*In vivo* transfection activity of 4-arm PEG-SSPHIS copolymer was studied by intravenous injection of the copolymer-based polyplexes in nude mice bearing HepG2 tumor using plasmid DNA encoding luciferase gene. Biodistribution of luciferase gene expression was detected after one-day injection (Figure 6). Herein, the polyplexes at the mass ratio of 12/1 were applied for *in vivo* transfection due to their improved colloidal stability as well as efficient *in vitro* transfection activity. It was found that the polyplexes afforded the highest gene expression in the tumor compared to other tissues and the gene expression was ~2 times higher than that yielded by the polyplexes of PEI as a control. It is known that nanoscale polyplexes are prone to accumulation in leaky tumor tissue due to enhanced permeability and retention (EPR) effect. In addition, the polyplexes were capable of mediating potent gene transfection under an acidic condition, as shown in transfection study *in vitro* (Figure 5). These reasons may favorably contribute to high gene expression in the tumor. Four-arm PEG-SSPHIS copolymer also induced detectable gene expression in the normal organs such as liver, spleen and kidney, giving a hint that its polyplexes were partially enriched in these organs. The accumulation of the polyplexes in liver and spleen may be due to opsonization process of slightly positively-charged polyplexes (~+9 mV, Figure 3d), where immune antibodies in the bloodstream actively interact with the polyplexes and they are then recognized by the phagocytic system. However, it should be noted that the polyplexes of 4-arm PEG-SSPHIS yielded significantly lower transgene expression in spleen and lung as compared to those of PEI at the mass ratio of 1/1. The high surface charge of PEI-based polyplexes (~+30 mV [13]) may contribute to their active opsonization and subsequent accumulation in the spleen. Moreover, PEI-based polyplexes induced transgene expression mainly in the lung because its unstable polyplexes tend to form large-sized aggregates which are mainly accumulated in the abundant capillary network of lung [26]. By contrast, the polyplexes of 4-arm PEG-SSPHIS had an improved colloidal stability in physiological conditions (Figure 3b), which may contribute to minimized enrichment in the lung and thus low transgene expression. Taken together, these results indicate that 4-arm PEG-SSPHIS copolymer may serve as an effective vector for intravenous gene delivery.

## 3. Experimental Section

### 3.1. Materials

4-Arm poly(ethylene glycol) (*M_W_* = 10 kDa, 4-arm PEG), histamine (HIS), cystamine bisacrylamide (CBA), glutathione (GSH), triethylamine (TEA), branched polyethylenimine (PEI, *M_W_* = 25 kDa) were ordered from Sigma-Aldrich (Shanghai, China). Fetal bovine serum (FBS), Dulbecco’s Modified Eagle Medium (DMEM) and phosphate buffered saline (PBS) were ordered from GIBCO (Shanghai, China). The plasmid, pCMV-eGFP and pCMV-Luc were purchased from plasmid factory (Bielefeld, Germany). Luciferase assay kit and cell lysis buffer were ordered from Promega (Beijing, China).

PEG vinyl sulfone was synthesized as reported previously [27]. The conversion degree of terminal group of PEG vinyl sulfone was calculated to be 96% from its ^1^H NMR spectra (CDCl_3_) by comparing the integrals of the signals at δ 3.6–3.7 (methyl protons of PEG) and δ 6.8 (proton of vinyl sulfone group). ^1^H NMR: δ 3.6–3.7 (OCH_2_CH_2_), 6.1 (CH_2_=CH), 6.4 (CH_2_=CH) and 6.8 (CH2=CH).

### 3.2. Synthesis of 4-Arm PEG-block-poly(disulfide histamine) (4-Arm PEG-SSPHIS) Copolymer

Four-arm PEG-SSPHIS copolymer was obtained by two-step procedure. First, poly(disulfide histamine) oligomer with amino terminal groups was synthesized. The oligomer was then reacted with 4-arm PEG vinyl sulfone to yield 4-arm PEG-SSPHIS copolymer. In brief, SSPHIS oligomer with average-number polymerization degree *X_n_* = 9 was prepared by stirring a mixture of CBA (0.864 g, 3.33 mmol) and an excess amount of HIS (0.463 g, 4.167 mmol) in methanol/water (1.6 mL, 4/1 *v*/*v*) in a brown reaction flask at 45 °C in the dark under N_2_ atmosphere. Once the reaction had proceeded for 5 days, 4-arm PEG vinyl sulfone (50 mg) was added into the flask via six portions and the stirring continued for another 3 days. Finally, the resulting solution was diluted with water to ~30 mL, acidified with 4 M HCl to pH~4, and purified by exhaustive dialysis (3 × 5 L, 15,000 cut-off) with deionized water (pH~4). 4-arm PEG-SSPHIS copolymer was isolated as water powder (yield: 102 mg, 93%).

### 3.3. Chemical and Biophysical Characterization

^1^H NMR (300 MHz) spectra were recorded on a Varian Inova spectrometer (Varian, Palo Alto, CA, USA). The signals of solvent residues were used as reference and were set at δ 4.79 for D_2_O.

The polyplexes of 4-arm PEG-SSPHIS at different mass ratios from 6/1 to 24/1 were prepared by gently mixing the polymer solution (800 μL, different concentrations in 20 mM HEPES buffer at pH 7.4) with DNA solution (200 μL of 75 μg/mL in 20 mM HEPES buffer at pH 7.4), followed by vortexing for 5 s and then incubation at room temperature for 30 min. Particle size and surface charge of the polyplexes were measured at 25 °C with Nanosizer NS90 (Malvern Instruments, Malvern, UK). To evaluate colloidal stability, saline solution was added to set a final salt concentration of 130 mM. Then, particle size of the polyplexes was measured at different time intervals (0.5, 4 and 24 h).

### 3.4. Agarose Gel Electrophoresis Assay

The polyplexes at different mass ratios were prepared by gently mixing the polymer solution (10 μL, different concentrations in 20 mM HEPES at pH 7.4) with DNA solution (10 μL, 80 μg/mL in 20 mM HEPES at pH 7.4), followed by incubation for 30 min at room temperature. Then, 10 μL of HEPES buffer (as control) or the buffer having GSH was added to set final GSH concentration of 10 mM, and the mixtures were incubated for another 30 min. After the addition 6× loading buffer (Fermentas, St. Leon-Rot, Germany), the mixture (10 μL) was loaded in the wells of a 0.7% agarose gel containing ethidium bromide (0.7 μg/mL). DNA was visualized by Tanon Gel Image system (Tanon, Shanghai, China) after running the gel electrophoresis at 100 mV for 30 min.

### 3.5. Cell Culture and Gene Transfection in Vitro

COS-7, NIH 3T3, MCF-7 and HepG2 cells (ATCC) were cultured in DMEM medium, containing 10% FBS and 100 U/mL penicillin and streptomycin (GIBICO, Shanghai, China). Transfection experiments were performed with pCMV-GFP or pCMV-Luc plasmid. The cells were plated in a 6-well plate (1 × 10^5^ cells/well) and cultured in 2 mL of completed medium for at least 24 h until 60%–70% cell confluence. Then, they were washed with 1× PBS buffer and incubated in the medium with or without 10% FBS for gene transfection. In a typical transfection protocol, the polyplexes of 4-arm PEG-SSPHIS at different mass ratios were prepared. Afterwards, the cells were transfected with the polyplexes (2 μg DNA) for 4 h at 37 °C in a 5% CO_2_-containing atmosphere. Next, the medium was replaced with completed medium and the cells were further incubated for another 44 h. A transfection formulation with B-pEI, prepared at a mass ratio of 1/1, was also applied as a positive control. All the transfection experiments were done in triplicates [13,14].

The influence of chloroquine (CQ) on the transfection efficiency of the polyplexes of 4-arm PEG-SSPHIS at the polymer/DNA mass ratio of 12/1 was examined by using the same transfection protocol as mentioned above. In this case, MCF-7 cells were incubated with the polyplexes in the presence of CQ (final conc. 100 μM) for 4 h and were allowed to incubate further for a total of 44 h.

Next, the cell viability was evaluated by AlamarBlue assay accordingly our previous protocol. In detail, the cells (including untreated cells, *i.e*., cells not exposed to transfection) were washed with fresh 1× PBS buffer and co-incubated with fresh-made 2 mL of 1× Alamar Blue-DMEM media (*i.e*., 10-fold diluted Alamar Blue solution in DMEM full medium) for 4 h. Afterward, 200 μL of the media from each well (and also 1× Alamar Blue-DMEM media as a blank) were transferred to a 96-well plate for fluorescence recording. The fluorescence intensity was recorded by a fluorescence plate reader (Perkin Elmer LS50B, Thermo Fisher Scientific, Hudson, SD, USA) with an excitation and emission wavelengths of 545 and 590 nm, respectively. Cell viability was calculated according to the equation: Cell viability (%) = (*F*_s_ − *F*_0_)/(*F*_c_ − *F*_0_) × 100, in which *F*_s_, *F*_c_, and F_0_ are fluorescence density of the medium of transfected cells, the medium of untreated cells, and 1× Alamar Blue-DMEM medium as a blank, respectively. The calculated value for untreated cells as a control was taken as 100% cell viability.

Transfection efficiency assay was determined by flow cytometry (BD) to quantify the percentage of GFP expressing cells. In brief, after Alamar Blue assay, the Alamar Blue-DMEM media was removed and the cells were washed with 1× PBS buffer, trypsinized and transferred to sterile tubes followed by centrifugation at 300× *g* for 5 min. The supernatant was poured off, and the cells were re-suspended in 0.4 mL of 1× PBS. Fluorescence for GFP was detected using a flow cytometry with an excitation and emission wavelengths of 488 and 535 nm, respectively. The cytometry was calibrated with a negative control (untreated cells) to identify blank transfection efficiency. The percentages of transfected cells were quantified from a gated viable population of 10,000 cells.

### 3.6. Cytotoxicity Assay of 4-Arm PEG-SSPHIS

COS-7 cells (10^4^ cells/well) were seeded in a 96-well plate. As the confluence reached 70%~80%, the medium was replaced with serum-free medium. The cells were then exposed in 4-arm PEG-SSPHIS at polymer concentrations in the range from 5–600 μg/mL. After 4 h incubation, the cells were cultured again in completed medium for another 44 h. Cell viability was determined by Alamar Blue assay, accordingly to the protocol mentioned above.

### 3.7. In Vivo Gene Transfection in HepG2 Tumor Bearing in Nude Mice

Animal experiments were officially approved by the Institutional Animal Care and Use Committee of Tongji University. Four to six-week-old male Balb/c nude mice bearing HepG2 tumor (~6 × 6 mm) were intravenously administrated via tail vein with 300 μL of the polyplexes of 4-arm PEG-SSPHIS at the mass ratio of 12/1 containing 30 μg of pCMV-Luc and 5% glucose. After 24 h, the mice were sacrificed and tumor, liver, lung, spleens, and kidneys were harvested, washed with 1× PBS and homogenized in passive lysis buffer prepared by mixing 1 mL of 5× passive lysis buffer (Promega, Beijing, China) 200 μL of 50 mM phenylmethylslfonyl fluoride (PMSF) in methanol, and 100 μL of protease inhibitor (Sigma, Shanghai, China). The homogenate was centrifuged at 12,000× *g* for 10 min at 4 °C. Then, 10 μL of the supernatant was mixed with 100 μL of luciferase assay reagent (Promega, Beijing, China) to detect luciferase expression with a luminometer (Thermo Scientific, Hudson, SD, USA). Total protein concentration in collected supernatant was determined by BAC Protein Assay kit (Invitrogen, Shanghai, China) and normalized using a standard BSA curve. Data were given as mean values (standard deviations) of four experiments and expressed as relative light unit (RLU)/mg protein [28].

### 3.8. Statistical Analysis

Comparisons between two samples were performed with the student’s *t*-test. Differences were considered to be statistically significant at *p* < 0.05.

## 4. Conclusions

We have demonstrated that 4-arm poly(ethylene glycol)-b-poly(disulfide histamine) copolymer can be readily obtained by Michael-type addition reaction. The polyplexes of the copolymer possess a few favorable gene delivery properties such as nanoscale size, colloidal stability, intracellular gene release and pH-responsive surface charges at acidic pH values. This copolymer induces efficient transfection activity in MCF-7 and HepG2 cancer cells specifically in acidic tumor microenvironment, meanwhile following with low cytotoxicity. This copolymer also causes detectable transgene expression mainly in HepG2-tumor rather than normal organs. Thus, 4-arm poly(ethylene glycol)-b-poly(disulfide histamine) copolymer reveals high potential for safe and efficient gene delivery.

## References

[1] Anderson W.F. (1998). Human gene therapy. Nature.

[2] Thomas C.E., Ehrhardt A., Kay M.A. (2003). Progress and problems with the use of viral vectors for gene therapy. Nat. Rev. Genet.

[3] Verma M., Somia N. (1997). Gene therapy—Promises, problems and prospects. Nature.

[4] Li S., Huang L. (2000). Non-viral gene therapy: Promises and challenges. Gene Ther.

[5] Merdan T., Kopecek J., Kissel T. (2002). Prospects for cationic polymers in gene and oligonucleotide therapy against cancer. Adv. Drug Deliv. Rev.

[6] Anwer K., Rhee B.G., Mendiratta S.K. (2003). Recent progress in polymeric gene delivery systems. Crit. Rev. Ther. Drug Carr. Syst.

[7] Pack D.W., Hoffman A.S., Pun S., Stayton P.S. (2005). Design and development of polymers for gene delivery. Nat. Rev. Drug Discov.

[8] Boussif O., Lezoualch F., Zanta M.A., Mergny M.D., Scherman D., Demeneix B., Behr J.P. (1995). A versatile vector for gene and oligonucleotide transfer into cells in culture and *in vivo*: Polyethylenimine. Proc. Natl. Acad. Sci. USA.

[9] Luten J., van Nostruin C.F., de Smedt S.C., Hennink W.E. (2008). Biodegradable polymers as non-viral carriers for plasmid DNA delivery. J. Control. Release.

[10] Cheng R., Feng F., Meng F.H., Deng C., Feijen J., Zhong Z.Y. (2011). Glutathione-responsive nano-vehicles as a promising platform for targeted intracellular drug and gene delivery. J. Control. Release.

[11] Lin C., Engbersen J.F.J. (2009). The role of the disulfide group in disulfide-based polymeric gene carriers. Expert Opin. Drug Deliv.

[12] Son S., Namgung R., Kim J., Singha K., Kim W.J. (2012). Bioreducible polymers for gene silencing and delivery. Acc. Chem. Res.

[13] Lin C., Zhong Z., Lok M.C., Jiang X., Hennink W.E., Feijen J., Engbersen J.F.J. (2007). Novel bioreducible poly(amido amine)s for highly efficient gene delivery. Bioconjate Chem.

[14] Lin C., Blaauboer C.-J., Timoneda M.M., Lok M.C., van Steenbergen M., Hennink W.E., Feijen J., Zhong Z.Y., Engbersen J.F.J. (2008). Bioreducible poly(amido amine)s with oligoamine side chains: Synthesis, characterization, and structural effect on gene delivery. J. Control. Release.

[15] Brumbach J.H., Lin C., Yockman J., Kim W.J., Blevins K.S., Engbersen J.F.J., Feijen J., Kim S.W. (2010). Mixtures of poly(triethylenetetramine/cystamine bisacrylamide) and poly(triethylenetetramine/cystamine bisacrylamide)-g-poly(ethylene glycol) for improved gene delivery. Bioconjate Chem.

[16] Wiethoff C.M., Middaugh C.R. (2003). Barriers to nonviral gene delivery. J. Pharm. Sci.

[17] Nishikawa M., Huang L. (2001). Nonviral vectors in the new millennium: Delivery barriers in gene transfer. Nucleic Acid Res.

[18] Plank C., Mechter K., F.C.S., Wagner E. (1996). Activation of the complement system by synthetic DNA complex: A potential barrier for intravenous gene delivery. Hum. Gene Ther.

[19] Lin C., Engbersen J.F.J. (2011). PEGylated bioreducible poly(amido amine)s for non-viral gene delivery. Mater. Sci. Eng. C.

[20] Mishra S., Webster P., Davis M.E. (2004). PEGylation significantly affects cellular uptake and intracellular trafficking of non-viral gene delivery particles. Eur. J. Cell Biol.

[21] Hatakeyama H., Akita H., Harashima H. (2011). A multifunctional envelope type nano device (MEND) for gene delivery to tumours based on the EPR effect: A strategy for overcoming the PEG dilemma. Adv. Drug Deliv. Rev.

[22] Tian L., Bae Y.H. (2012). Cancer nanomedicines targeting tumor extracellular pH. Colloids Surf. B.

[23] Cheng J., Zeidan R., Mishra S., Liu A., Pun S.H., Kulkarni R.P., Jensen G.S., Bellocq N.C., Davis M.E. (2006). Structure-Function correlation of chloroquine and analogues as transgene expression enhancers in nonviral gene delivery. J. Med. Chem.

[24] Knorr V., Allmendinger L., Walker G.F., Paintner F.F., Wagner E. (2007). An acetal-based PEGylation reagent for pH-Sensitive shielding of DNA polyplexes. Bioconjate Chem.

[25] Knorr V., Ogris M., Wagner E. (2008). An acid sensitive ketal-Based polyethylene glycol-oligoethylenimine copolymer mediates improved transfection efficiency at reduced toxicity. Pharm. Res.

[26] Zou S.M., Erbacher P., Remy J.S., Behr J.P. (2000). Systemic linear polyethylenimine(L-PEI)-mediated gene delivery in the mouse. J. Gene Med.

[27] Lin C., Zhao P., Li F., Guo F., Li Z., Wen X. (2010). Thermosensitive in situ-forming dextran-pluronic hydrogels through Michael addition. Mater. Sci. Eng. C.

[28] Lin C., Song Y., Lou B., Zhao P. (2014). Dextranation of bioreducible cationic polyamide for systemic gene delivery. Bio-Med. Mater. Eng.

